# Fostering Workforce Wellness: Insights from Nurse Managers and Early Childhood Educators

**DOI:** 10.3390/healthcare13050487

**Published:** 2025-02-24

**Authors:** Dominique Charlot-Swilley, Sabrina Zuskov, Latisha Curtis, Stephanie Mitchell, Elva Anderson

**Affiliations:** 1 Department of Pediatrics, Georgetown University School of Medicine, 2115 Wisconsin Avenue, N.W., 6th Floor, Washington, DC 20007, USA; sm3878@georgetown.edu; 2 Department of Psychiatry, Georgetown University Medical Center, Washington, DC 20007, USA; szuskov@gmail.com (S.Z.); lc1229@georgetown.edu (L.C.); 3 Department of Psychiatry & Behavioral Sciences, Children’s National Hospital, Washington, DC 20010, USA; eanderso@childrensnational.org

**Keywords:** mindfulness-based intervention, burnout, compassion fatigue, wellness champion, organizational culture, leadership engagement, workforce wellbeing, early childhood educators, nurse managers

## Abstract

**Background/Objectives**: The COVID-19 pandemic has highlighted the challenges faced by essential professionals, such as nurse managers and early childhood educators (ECEs), who grapple with heightened stress, burnout, and compassion fatigue. In response, the *Compassion*, *Practice*, *Relationships*, *and Restoration* (CPR^2^) program was designed as a virtual, structured wellness intervention to address these pressing concerns among caregiving professionals. **Methods**: A mixed-methods study was employed to evaluate the feasibility, acceptability, and preliminary outcomes of CPR^2^, implemented across two cohorts: nurse managers in a pediatric hospital and early childhood educators (ECEs) serving equity-deserving communities. Participants completed electronic surveys at pre- and post-test assessing mindfulness, professional quality of life, healthy lifestyle behaviors, and perceived social cohesion. One month post-program, focus groups were conducted using a facilitation guide to evaluate program elements, including group structure, expectations, discussion themes, and sustainability. **Results**: Quantitative findings suggest that while nurse managers experienced significant reductions in compassion fatigue, along with improvements in mindfulness and perceived cohesion, ECEs exhibited stable levels of stress and burnout. Both cohorts reported enhanced sleep quality, emphasizing the program’s potential to foster critical aspects of wellbeing. Qualitative participant feedback highlighted the importance of organizational readiness, leadership engagement, and program flexibility for successful implementation. **Conclusions**: The study highlights the need for tailored, context-sensitive wellness interventions that recognize the unique challenges faced by different caregiving roles. It also emphasizes the potential for sustained impact when wellness initiatives are integrated into the organizational culture, further reinforcing the importance of ongoing commitment to workforce wellbeing in high-stress environments.

## 1. Introduction

The COVID-19 pandemic and its aftermath illuminated the immense pressures and systemic challenges faced by essential professionals, including frontline healthcare workers such as nurses and nurse managers [1], while also highlighting the significant challenges experienced by early childhood educators (ECEs) [2]. During the pandemic, nurses’ responsibilities expanded significantly as they faced unprecedented demands in providing care for patients, managing elevated levels of stress, and safeguarding their own health [3,4,5]. A 2020 survey revealed that nearly two-thirds of nurses (62%) experience burnout, with the highest rates among younger nurses—69% of those under twenty-five reported feeling burned out [4]. Burnout, as defined by the World Health Organization, is a syndrome resulting from chronic workplace stress that has not been successfully managed, characterized by emotional exhaustion, depersonalization, and a reduced sense of personal accomplishment [6]. This definition emphasizes the impact of burnout specifically related to the work context and as an emotional indicator of professional wellbeing differentiating it from other mental health conditions or experiences of stress outside of the workplace.

To further highlight the growing challenges of COVID-19, a longitudinal study by the American Organization for Nursing Leadership found that the pandemic has intensified stress among nurse leaders, further highlighting the increasing challenges associated with burnout [7]. Analyzing data from over 2500 nurse leaders, the study found that moral distress, burnout, and fatigue were pervasive across all levels of leadership. Notably, one in four nurse managers reported that they are not emotionally healthy. Emotional health and wellbeing were a primary concern of nurse leaders (45%), followed by the growing and emerging challenge of nurse recruitment and retention (34%). A cross-sectional study assessing psychosocial job stress and associated health risks among healthcare professionals reported that 93% of nurses experienced elevated stress levels [8]. These findings align with global reports on the prevalence of work-related stress among nurses [9,10,11,12].

Similar to findings on burnout in nurse leaders, younger nurses and those with 2 to 5 years of professional experience are most at risk for experiencing compassion fatigue [13]. Compassion fatigue refers to the physical, emotional, and psychological impact of helping others who are in distress, often described as the “cost of caring” for professionals in caregiving roles [14] and continuously “witnessing and absorbing the suffering and challenges of others”, [15]. Burnout develops over time due to chronic stress in the workplace, while compassion fatigue can occur more suddenly and is specifically linked to the physical strain of caring for others and can lead to a diminished capacity for empathy and emotional engagement. Compassion fatigue among younger or less experienced nurses is associated with higher turnover rates, decreased retention, absenteeism, and an increased likelihood of leaving the nursing profession [13,16]. Presenteeism, in contrast to absenteeism, refers to the phenomenon where a nurse or healthcare provider is physically present at work despite being unfit for duty due to illness or job-related stressors. Nurses demonstrate the highest rates of presenteeism within the workforce, which has been associated with adverse patient outcomes and diminished patient safety [17]. Secondary traumatic stress, a component of compassion fatigue, represents another significant consequence of prolonged exposure to others’ trauma that is associated with sleep disturbances and other indicators of poor emotion coping [13,18,19].

While the prevalence of work-related stress among nurses is well documented, early childhood educators (ECEs) face similarly alarming challenges. Tasked with the immense responsibility of nurturing young children during critical developmental stages, ECEs navigate a combination of high emotional demands, limited resources, and persistent undervaluation of their profession [20,21]. Factors such as low compensation, limited access to mental health support, and chronic workforce shortages place significant strain on ECEs, contributing to high levels of burnout and turnover [20,21]. Occupational burnout has been found to be as high as 56% among ECEs [22], which affects social and mental health and is linked to higher rates of chronic disease and premature mortality [23]. The unique challenges faced by ECEs are compounded by the dual role they play as both caregivers and educators. Unlike in many other professions, ECEs must constantly balance the emotional labor of nurturing relationships with children and families with the pedagogical responsibilities of fostering early learning and development. Research highlights that ECEs often serve as the first line of support for families navigating economic instability, food insecurity, and trauma, further amplifying their workload and emotional strain [24]. Moreover, the lack of systemic support for ECEs contributes to a cyclical pattern of stress. For instance, low staff-to-child ratios and high turnover rates often force remaining educators to take on added responsibilities, creating an unsustainable workload. This is particularly evident in under-resourced early learning centers, where limited access to professional development, mental health support, and classroom resources disproportionately impacts educators of color and those serving communities most impacted by historical inequities [25].

The systemic undervaluation of ECE as a profession is deeply rooted in historical and structural inequities. Early childhood education, predominantly staffed by women—and disproportionately by women of color—has long been associated with care work rather than formal education, leading to persistent wage gaps and inadequate professional recognition [26]. This devaluation is not a recent phenomenon; it mirrors historical patterns where caregiving roles, primarily held by women of color affected by systemic inequality, have long been undervalued. The historical treatment of wet nurses provides a compelling parallel to the challenges faced by ECE professionals today. Wet nurses, often women who experience the burden to systemic oppression, performed critical caregiving roles, yet their labor was undervalued, undercompensated, and regarded as menial despite its profound impact on child development [27]. Although the critical nature of this labor for child survival and development is undeniable, it has often been framed as low-status, feminized labor, performed out of economic necessity rather than professional skill [28,29]. Similarly, ECE professionals occupy a pivotal role in shaping early developmental outcomes, yet they face systemic devaluation rooted in the same gendered and racialized perceptions of caregiving as unskilled or secondary labor. Both groups experience a form of gendered exploitation, as caregiving roles have traditionally been seen as women’s work and thus undervalued in comparison to other professions. This gender bias reinforces the societal tendency to neglect the physical and emotional demands of caregiving roles, despite their essential contribution to societal wellbeing [30] and child development [31].

By understanding the deep-rooted societal attitudes that have long devalued caregiving work predominately performed by women of color [25,26], it becomes possible to better advocate for policies and practices that elevate the status, compensation, and working conditions of ECE professionals. This includes re-examining societal values around care work and implementing systemic changes to ensure that those who perform this vital labor are adequately compensated and respected [32]. Research indicates that ECEs experience high levels of emotional labor and burnout due to societal undervaluation of their work [30,31,32,33]. Just as wet nurses in the past faced exploitation and neglect in their professional environments, ECEs today often contend with low wages, limited professional recognition, and a lack of institutional support, all contributing to diminished job satisfaction [33,34,35]. The economic disparities in early childhood education further highlight the systemic challenges these professionals face.

ECEs serve young children across diverse settings, including public programs like Universal Pre-Kindergarten and Head Start, as well as private environments such as faith-based, home-based, and center-based childcare [36]. Despite playing a critical role in fostering early brain development and future academic success, the median pay for ECEs remains significantly lower than that of educators in K-12 systems, often leaving them below the poverty line [37]. These economic disparities not only exacerbate stress among educators but also contribute to the high turnover rates [33,34] that destabilize early learning environments. Furthermore, federal and state policies, while designed to promote high-quality care, can inadvertently place additional burdens on ECEs. Compliance with rigorous licensing standards, such as detailed documentation and ongoing assessments, often requires considerable time and administrative effort without corresponding support or compensation [33]. Educators frequently report spending unpaid hours outside of their classroom responsibilities to meet regulatory requirements, deepening their sense of being overworked and underappreciated [38]. These expectations, while essential for accountability, exacerbate the already challenging work conditions.

Both nurse managers and ECEs are professions rooted in caregiving and relational work, which inherently involves high levels of emotional labor. These roles require not only technical and professional expertise but also sustained empathy, adaptability, and fortitude in environments often characterized by limited resources, high stakes, and complex interpersonal dynamics. While the settings differ, both groups experience disproportionately high rates of burnout and compassion fatigue due to systemic undervaluation of their professions, chronic understaffing, and the emotional intensity of their responsibilities [15,39]. This dual focus calls attention to the urgent need for wellness initiatives tailored to professions where the intersection of emotional labor and systemic inequity often goes unaddressed, offering a deeper exploration of the shared challenges related to emotional labor, systemic inequities, and chronic stress.

Given these shared challenges coupled with the heightened emotional and mental health challenges brought on by the COVID-19 pandemic, the group-based *Compassion*, *Practice*, *Relationships*, *and Restoration* (CPR^2^) program was developed to support nurse managers and ECEs. This wellbeing initiative provides tools and strategies to help participants manage emotional distress, cultivate wellness practices, and strengthen their resources to manage stress and burnout, with the goal of enhancing their overall quality of life and capacity to thrive professionally. CPR^2^ incorporates a psychoeducational component designed to enhance participants’ understanding of stress, trauma, and emotional regulation while equipping them with evidence-based strategies to address these challenges effectively. Psychoeducation involves teaching individuals about the psychological and physiological effects of stress, burnout, and compassion fatigue, enabling them to identify their own triggers and responses. Participants are introduced to concepts such as the neurobiology of stress, the role of mindfulness in managing emotional distress, and the impact of self-compassion on professional wellbeing. Through structured learning sessions, they gain practical knowledge about coping mechanisms, boundary setting, and wellness practices.

Beyond the psychoeducational component, the program emphasizes evidence-based tools and strategies, such as mindfulness exercises, stress-reduction techniques, and micro-restorative practices, to directly address emotional distress, build stronger social connections, and support participants in managing stress, mitigating burnout, and fostering overall wellbeing. Micro-restorative practices refer to small, intentional actions or strategies that individuals can use throughout their day to restore emotional balance, reduce stress, and promote wellbeing. These practices are often quick, accessible, and can be integrated into daily routines, helping individuals manage stress in the moment. Examples include deep breathing, mindful pauses, positive affirmations, or brief moments of self-reflection [40,41,42,43,44] The goal of micro-restorative practices is to offer immediate support to help individuals recover from emotional or mental fatigue, cultivating wellness and enhancing emotional awareness without requiring considerable time or resources.

Research consistently shows that group-based interventions are highly effective in achieving these goals [45,46,47]. In healthcare and educational settings, group programs help mitigate stress, reduce burnout, and foster social support by promoting shared experiences and collective learning. For nurse managers, group programs enhance emotional intelligence, strengthen leadership skills, and facilitate peer learning, which collectively improve participants’ ability to navigate complex workplace challenges [48,49]. Similarly, in early childhood education settings, group-based approaches have been associated with reductions in stress and burnout, improved emotional regulation, and strengthened workplace connections through shared learning and mindfulness practices [50,51]. These findings highlight the dual value of peer support and practical strategies in mitigating professional stressors across healthcare and education sectors, ultimately fostering improved individual and organizational outcomes.

Building upon these insights, the CPR^2^ program is grounded in several key theories, each contributing to a comprehensive approach for managing stress and improving emotional wellbeing. Mindfulness-Based Stress Reduction (MBSR) is a central component, focusing on mindfulness meditation and body awareness to reduce stress and enhance emotional regulation. MBSR cultivates present-moment awareness, helping individuals approach their experiences with acceptance and non-judgmental awareness, which has been shown to alleviate symptoms of anxiety, depression, and stress [52,53]. Research across disciplines such as psychology, neuroscience, and medicine provide substantial evidence that mindfulness affects attention [54], cognition [55], emotions [56], and behavior [57]. Workplace mindfulness, the extent to which individuals are mindful in their work setting, helps with improvements in task commitment, enjoyment of work [57,58] and social relationships [57]. As reported by Hulsheger et al. [58], positive workplace relationships can buffer the effects of workplace stressors, promote thriving in employees, foster better communication, and creativity. Researchers have also linked mindfulness to better communication, lower emotional stress, and positive change in perceptions of relationships [59,60].

Cognitive-Behavioral Theory (CBT) complements MBSR by focusing on identifying and altering negative thought patterns that influence emotions and behaviors. By reframing maladaptive thoughts, CBT strengthens emotional regulation and coping strategies, which is essential for preventing burnout and managing stress [61,62]. By identifying and challenging negative or maladaptive thought patterns, CBT helps individuals alter their emotional responses and behaviors, ultimately improving mental health and stress management. It is particularly effective for conditions like anxiety, depression, and stress, making it valuable in high-stress professions such as healthcare and early childhood education (ECE). In healthcare settings, research has shown that CBT-based interventions can significantly reduce burnout, anxiety, and stress among nurses, doctors, and other providers. A study by Sjöberg et al. [62] found that CBT interventions significantly reduced work-related stress and emotional exhaustion in healthcare professionals. A meta-analysis by Westwood et al. [63] demonstrated that CBT interventions significantly reduce emotional exhaustion and depersonalization among nurses, two core components of burnout. By addressing maladaptive thinking, CBT helps nurses regain a sense of purpose and efficacy in their work. Maslach and Leiter [64] also found that CBT can address the cognitive distortions contributing to burnout, helping individuals regain a sense of efficacy and reduce emotional exhaustion. Similarly, Masten et al. [65] highlighted how CBT techniques help educators manage stress by reframing negative thoughts related to their workload and student interactions. Li et al. [66] also noted that CBT can provide tools for ECEs to better handle challenges like low pay, high demands, and limited support, contributing to better mental health and job satisfaction. These studies suggest that CBT not only reduces stress but also helps improve the coping strategies of individuals working in high-pressure environments.

Lastly, Healing-Centered Engagement (HCE) [67,68] represents a holistic and integrative approach to addressing trauma and stress, shifting beyond trauma-informed care to emphasize wellness, agency, and connection. HCE builds upon existing frameworks such as trauma-informed care models that emphasize safety, trust, and empowerment [69] as well as culturally relevant practices [70] while also drawing on the importance of focusing on posttraumatic growth, where individuals, through community and healing practices, can experience positive transformations after trauma [71]. Rooted in a strengths-based perspective, HCE emphasizes building individual and collective assets rather than dwelling on deficits or pathologies. This approach is culturally and contextually grounded, acknowledging the role of systemic and historical influences on trauma and wellbeing while centering dignity and cultural affirmation in interventions. It views healing as a comprehensive process that integrates emotional, physical, mental, and spiritual wellbeing. Furthermore, it emphasizes individuals’ active participation in the healing journeys by fostering self-awareness, advocacy, and opportunities for meaningful growth and restoration.

Building on these theoretical frameworks, the CPR^2^ program seeks to evaluate the feasibility, acceptability, and initial impact of the CPR^2^ wellbeing program, aiming to examine its effectiveness in addressing the unique stressors faced by nurse managers and ECEs while fostering positive outcomes for both individuals and the organizations they serve. The program hypothesizes that participants will integrate mindfulness practices into their daily lives and adopt healthier behaviors, leading to measurable reductions in compassion fatigue and burnout. This paper presents findings from mixed-methods pilot studies of CPR^2^, delivered to two cohorts—nurse managers and ECEs—caring for families and children during the COVID-19 pandemic.

## 2. Methods

### 2.1. Study Design

CPR^2^ was pilot-tested in two distinct workplace contexts. Cohort 1 ran from February 2021 to April 2021 with nurse managers. Cohort 2 ran from June 2021 to October 2021 with ECEs from two different centers, one serving a homeless population and the other victims of domestic violence. Both cohorts engaged in a 10-session virtual, structured program, convening once a week, which was designed to support their wellbeing and professional development. The program was run virtually due to COVID-19 Pandemic and restrictions on in-person gathering. For this pilot, a mixed-methods evaluation was conducted and approved by the Children’s National Hospital and Georgetown University Internal Review Boards (IRB). Quantitative data were collected through an electronic self-reported survey administered during the first and last session. These pre- and post- intervention data were compared to examine whether there were any changes in mindfulness practices, healthy behaviors, compassion fatigue and burnout after program participation. One month after each cohort’s program completion, a focus group was conducted to gather participant feedback on the program.

### 2.2. Program Sites

All sites were located within Washington D.C. and were selected as a convenience sample based on existing partnerships with pilot developers. The early childhood education sites included head start programs specifically serving children from birth to five years old. These programs were chosen for their mid-sized capacity, accommodating between 65 and 100 children per site. These sites serve high-need populations, including families experiencing homelessness and domestic violence, which profoundly influence both the developmental needs of the children, and the work demands placed on educators. The focus on these sites reflects the study’s objective to examine staff wellbeing in a field characterized by elevated stressors and challenges.

The hospital site, where nurse managers participated, was a private, non-profit, urban children’s hospital located in Washington D.C. This institution was selected due to the researchers’ professional affiliation, which facilitated access to participants and relevant resources. The hospital’s specialized focus on pediatric care provided a unique opportunity to examine staff wellbeing in a high-stress, service-intensive healthcare setting, aligning with the study’s aim to explore wellbeing in environments with heightened caregiving demands.

### 2.3. Participants

For Cohort 1, all nurse managers were sent an electronic message inviting them to participate in a 10-week, hour-long wellness group for which they would receive Continuing Education Units (CEUs) per session. Participants had to attend 8 out of 10 sessions to be eligible for CEUs. The first twenty nurse managers to sign up, gave informed consent and participated in the intervention. One participant did not complete the pre-test survey, so their data is not reported in this analysis (n = 19).

For Cohort 2, ECEs were invited to participate through flyers distributed by center leadership, which outlined the CPR^2^ program and invited staff to learn more. The flyer included an option for interested participants to share their contact information, signaling their willingness to engage with the research team. The research team then followed up with these individuals to provide further details about the study and administer consent forms for those who chose to participate. A member of the research team organized virtual group appointments with potential participants to provide a comprehensive overview of the CPR^2^ program, discuss the program schedule, and clarify the consent and research procedures. Participants in Cohort 2 were compensated a total of USD 500 for their involvement. Of this, USD 35 was provided for each data collection point, totaling USD 105. The remaining USD 395 was distributed directly by the participating sites. Participants were assured that declining to participate in the research would not affect their role at the center or their participation in the CPR^2^ program. Two participants from Cohort 2 did not complete the post-test survey after three requests and were subsequently excluded from the analyses (n = 17).

### 2.4. Intervention

CPR^2^ is a virtual small group program of 10 weekly sessions that utilizes evidence-based activities designed to enhance skill building and social connectedness. It was facilitated by the developer, a trained clinical psychologist and the program’s research assistant, who has a background in psychology, aiding with participant engagement and ensuring smooth session facilitation. The groups were facilitated virtually to ensure accessibility and convenience for all participants, enabling them to engage in the intervention regardless of work location. The topics covered included an introduction to mindfulness and its connection to overall wellbeing, followed by sessions that explored breathwork, goal setting, cognitive and emotional wellbeing, and stress management. The program also addressed the specific challenges of coping with the impact of COVID-19, physical wellbeing through sleep, nutrition, and movement, as well as fostering social connections and practicing self-compassion. The final sessions integrated the practice of mindfulness with kindness to others, providing participants with a comprehensive approach to personal and professional wellbeing.

Each CPR^2^ session adhered to a structured format, including a mood rating check-in, mindfulness opening, psychoeducational topic content, group reflection and discussion, reflective journaling practice and mood rating check-out. The topics covered in 10 sessions were as follows:

1. Mindfulness and CPR^2^ Introduction;
2. Mindfulness Breath and Goal Setting;
3. Mindfulness and Cognitive Wellbeing;
4. Mindfulness and Emotional Wellbeing: Stress;
5. Mindfulness and Emotional Wellbeing: COVID and Beyond;
6. Mindfulness and Physical Wellbeing: Sleep and Nutrition;
7. Mindfulness and Physical Wellbeing: Physical Activity and Movement;
8. Mindfulness and Social Wellbeing;
9. Mindfulness and Self Compassion;
10. Mindfulness and Compassion to Others: Pulling It All Together.

The objectives of each session were tailored to the specific topic area and included a mindfulness activity aimed to improve general wellbeing or address a relevant concern or symptom discussed in that session. Session 1 served as a welcome and introduction and included the exploration of understanding of mindfulness practices, the demonstration of a wellness wheel activity, and the brainstorming and implementation of a self-care plan. Session 2 covered discussion and support around goal setting as well as the establishment of accountability partners. The accountability partner was self-selected within each cohort at the respective sites, allowing participants to connect, provide mutual support, and share lessons learned. This peer support structure aims to create a lasting community, reinforcing the principles of the program and facilitating continued engagement beyond the formal sessions.

The sessions that followed covered various content areas including psychoeducation around stress and trauma, self-compassion, and multiple topics and activities within physical, emotional, cognitive, and social wellbeing. Both emotional and physical wellbeing sessions were split into two sessions in order to incorporate the mindful movement, sleep, and nutritional aspects of physical wellness. Throughout these sessions, mindfulness components included grounding activities, song, meditation, movement, reading (poems and virtue cards), and body scans. At the end of each session, the leader shared a reflective journaling prompt related to the content of the session and encouraged participants to complete the prompt, document their practice, and share their experience with the group in the following session. The final session served as a review of all topics covered and facilitated a discussion about the topics, new relationships, lessons, or mindfulness activities that resonated most with participants or had the greatest impact on their daily life. The full overview of the topics and sessions is provided in Appendix A.

Each session was designed to offer both theoretical knowledge and practical strategies, creating a balanced framework for stress management and overall health. Specifically, each 1 h CPR^2^ session began with a mood rating check-in accompanied by music, providing participants a moment to settle in, pause, and engage in self-reflection. Music was chosen at random, typically jazz or upbeat lo-fi music played via YouTube. This was followed by a mindfulness activity focused on enhancing self-awareness and cultivating self-compassion led by the facilitator. These activities included, but were not limited to, body scan meditation, breathing exercises, yoga and mindful stretching, movement meditation, affirmations, and loving-kindness meditation. Psychoeducational content was then presented, integrated with information on micro-restorative practices. Integrating these small, restorative moments, allowed participants to practice them during the session and explore ways to incorporate them into their demanding work schedules and personal lives, helping to reduce stress, enhance focus and improve emotional regulation throughout the day.

### 2.5. Participant Observer

An onsite staff member served as a *Participant Observer* (PO) in the weekly group sessions. They did not receive formal training, but they engaged in regular debriefing and reflective practice sessions with the program developer to support their role and address any challenges or troubling findings. Participant observers offer valuable insights into participants’ lived experiences, allowing facilitators to adjust content and delivery to better meet the group’s needs [72,73,74]. This real-time feedback ensures the program stays flexible and relevant, enhancing engagement and outcomes [74]. The PO was intentionally positioned outside the formal power hierarchy, enhancing trust and accessibility for participants. For this reason, there was not a PO embedded in the sessions for the nurse managers, given her supervisory role; however, she served as an advocate, championing the program’s importance and supporting participants amidst the demands of their high-stress roles. She provided to the developer critical advisement on tailoring the program to address the specific needs of nurse managers. While the core program content remained consistent, her feedback allowed for the customization of examples to better align with the real-world challenges faced by this group. Regular touchpoints were maintained throughout the program to ensure its alignment with staff needs and expectations. Additionally, she played a pivotal role in facilitating equitable compensation by coordinating the provision of CEUs, addressing a professional priority for nurse managers who were unable to accept financial incentives due to employment agreements.

There were notable differences in the PO roles between the two early childhood centers. The PO from the domestic violence center left her position midway through the program and, due to the organization’s multi-site nature, had limited direct engagement with participants. In contrast, for the homeless violence site, the PO was an on-site mental health staff member, physically embedded within the site. Without holding a position of authority over participants, this role facilitated seamless integration into the group. This proximity allowed for consistent touchpoints with participants, fostering psychological safety and creating a supportive dynamic outside the facilitator–participant relationship. At both centers, participants were informed that the PO was available for continued support, both during and after the program’s conclusion. The PO’s role contributed to the program’s adaptability and responsiveness to participants’ needs. The observer went beyond traditional observation, actively participating in the group and attending all intervention sessions to fully integrate into the group’s dynamics and activities. This approach was an intentional design, with the PO actively participating in the group sessions as part of a strategy to transition into the role of Wellness Champion for the site. This dual role enabled the observer to gain a nuanced understanding of group dynamics and site-specific challenges from the participants’ perspective. Active participation also built trust and rapport, essential for the successful transition into the wellness champion role and for sustaining wellness practices post-study. Sustainability in this context refers to having the wellness champion support the continuation of wellness activities and practices within the site after the formal study concludes. The PO and the nurse manager’s advocate played a critical role in participant recruitment and engagement, actively encouraging enrollment and advocating for protected time to ensure participants could attend the program without conflict. This advocacy was essential to fostering a supportive environment that prioritized participant wellbeing and program accessibility. The observer was fully engaged throughout the entire 10-week intervention, ensuring comprehensive exposure to the group’s activities and evolution over time.

### 2.6. Survey Data Collection

In both cohorts, at the beginning of the first virtual session, participants were given a link to a Qualtrics online survey that included the questionnaires described below. When completing the pre-test survey, each participant created a unique user ID based on the year they began working at the hospital/ECE and the street number of their current home address. They reentered this ID number in the post-test survey (with the same instruments repeated) so that each participant’s response at pre-test and post-test could be matched for analysis.

Demographics. Participants were asked to report their age in years, current relationship status from eight fixed response options (e.g., never married/single, living with partner), which race/ethnicity they self-identify as from seven fixed response options (e.g., Black/African American, Hispanic or Latino), gender from six fixed response options (e.g., male, female, non-binary), number of people in household, language spoken at home from 3 fixed response options (English, Spanish, other [written in]), living situation from six response options (e.g., I own a home, I rent an apartment), highest level of education from eight response options, approximate annual pre-tax income from twelve fixed response options, family’s primary source of income from six fixed response options (e.g., salary/wage from your job, state or federal aid), years and months employed at current employer, and length of time working in the nursing or early education field from 8 fixed response options from “less than 1 year” to “more than 20 years”.

Mindfulness. For Cohort 1 (nurse managers), we administered the widely used and validated Five Facet Mindfulness Questionnaire (FFMQ) [75] derived from five previous mindfulness questionnaires. Participants rated how true 39 statements are about oneself on a 5-point scale from “never or very rarely true” (1) to “very often or always true” (5). Subscale scores are derived by reverse-scoring, as appropriate, and then summing responses across the 8-items for each subscale. *Observation* (α = 0.83) is how an individual sees, feels and perceives the internal and external world around them (e.g., “I notice how foods and drinks affect my thoughts, bodily sensations, and emotions.”). *Description* (α = 0.91) looks at how accurately an individual is able to describe their experiences, thoughts and feelings through words (e.g., “Even when I’m feeling terribly upset, I can find a way to put it into words.”). *Acting with Awareness* or “Act aware” (α = 0.87) measures an individuals’ tendency to bring their undivided attention and full awareness to their actions and experiences (e.g., “I find myself doing things without paying attention.”; reverse scored). *Non-judging of the Inner Experience* or “Nonjudge” (α = 0.87), refers to an individual not allowing an inner critical tone to overshadow their happiness or positive state of mind (e.g., “I think some of my emotions are bad or inappropriate and I shouldn’t feel them”.; reverse scored). *Non-reactivity of Inner Experience* or “Nonreact” (α = 0.75) is the individual’s ability to refrain from reacting to negative thoughts and emotions (e.g., “When I have distressing thoughts or images, I just notice them and let them go.”).

For Cohort 2 (ECEs), the Cognitive and Affective Mindfulness Scale (CAMS-R) [76], a 12-item self-report measure, was administered to assess participants’ mindfulness. The CAMS-R has been used in early childhood education settings to examine the influence of mindfulness practices on educators’ emotional regulation and overall wellbeing [37,38]. The measure’s tailored focus on early childhood educators was the main reasoning to use a different mindfulness scale compared to Cohort 1. Additionally, the measure has been utilized in other early childhood educator interventions, and its selection aligns with its use in related projects conducted by the researchers’ organization. This scale evaluates both cognitive and emotional dimensions of mindfulness, focusing on three core components: cognitive attention, emotional awareness, and acceptance. *Cognitive attention* refers to the ability to focus on the present moment (e.g., “I am able to pay close attention to one thing for a long period of time.”), while *emotional awareness* involves recognizing one’s emotions without becoming overwhelmed (e.g., “I can usually describe how I feel at the moment in considerable detail.”). *Acceptance* entails responding to thoughts and feelings with openness and without judgment (e.g., “I try to notice my thoughts without judging them.”). Participants rate each statement on a 4-point Likert scale (1 = rarely/never true, 4 = almost always true) based on their experiences over a specified time period, in this study [time frame]. Higher scores indicate greater mindfulness.

Lifestyle Questionnaire [77]. Participants were asked how many minutes they spent performing physical activity during the past 7 days, in a typical week how many minutes they spend in physical activity, how many servings of fruit they eat in a typical day, how many servings of vegetables they eat in a typical day, and how they would rate their sleep quality in the past week on a 4-point scale from ‘very bad’ to ‘very good’.

Professional Quality of Life Scale (ProQOL) [78]. This questionnaire asks respondents to rate how frequently, on a 5-point scale from ‘Never’ to ‘Very Often’, they experience the stated items in their current work situation over the past 30 days. The items load onto 3 distinct subscales, scores for which are derived by summing responses across the 10 items. *Compassion satisfaction* (α = 0.87) is about the pleasure one derives from being able to do work well (e.g., “I feel invigorated after working with those I [help].”). *Burnout* (α = 0.90) is feelings of hopelessness and difficulties in dealing with work or in doing one’s job effectively (e.g., “I feel overwhelmed because my workload seems endless.”). *Compassion fatigue* (α = 0.80), also referred to as secondary traumatic stress, is work-related, secondary exposure to extremely stressful events that causes symptoms such as being afraid, having difficulty sleeping, and having images of the upsetting event pop into your mind (e.g., “I find it difficult to separate my personal life from my life as a [helper]”.

Perceived Cohesion Scale [79]. Six questions ask respondents to rate the degree to which they agree with statements about their group (in this case the team of nurse managers) on a 7-point scale from ‘strongly disagree’ to ‘strongly agree’. Responses can be summed to calculate scores on the *Belonging* (e.g., “I see myself as part of this group.”) and *Morale* (e.g., “I am happy to be part of this group.”) subscales (3 items load onto each) which have shown good reliability in small group settings (α = 0.95 and α = 0.87, respectively). For Cohort 1, we administered both pre- and post-test measures of social cohesion to capture the change resulting from the mindfulness intervention. This allowed us to evaluate the baseline levels of social cohesion since the nurse managers where in different departments at CNH and directly assess how the intervention influenced this outcome. For Cohort 2, while the same measures were intended to be collected, logistical or resource constraints led to the decision to only administer the post-test measure of social cohesion. This decision was made to streamline data collection processes.

### 2.7. Qualitative Data Collection

All participants who completed the group were invited to participate in a one-hour focus group following the end of the 10-week program. At the final group session, the facilitator provided instructions to participants that an external research team member would be leading the focus group. Participation was voluntary and those who attended were compensated for their time and feedback. The focus group aimed to gather participants’ insights on their experiences during the CPR^2^ sessions and to collect feedback on potential improvements for the program in the future. Of the 20 nurse managers who participated in the program, 15 participated in the focus group. This low attendance was in part due to scheduling conflicts and emergencies in the hospital workplace. Similarly, 12 ECEs in the program participated in the focus group, with low attendance primarily due to the challenges of maintaining teacher-child ratio and the difficulty of leaving the classroom.

The focus group, led by an external research team member, encouraged the providers to share their feedback openly and honestly. Following research protocol, the focus group facilitator asked questions about the content, reflections around the structure of the program, benefits and challenges of the group, their overall experience, feedback on the weekly topics, and any personal takeaways after the 10 weeks. Additionally, specific questions were designed to explore areas such as group structure and formatting, expectations, helpful topics and activities covered, impact of mindfulness practices, reflective journaling, administrative support and sustainability. The focus group leader was instructed to prompt and probe on responses where appropriate in order to gain more insight into their experience. Questions from the focus group are below and full script can be found in Appendix B:

1. What was it like for you to participate in this program?
2. What are your thoughts regarding the length and frequency of the Wellness sessions?
3. Considering the weekly topics, any specific takeaways from the weeks?
4. Did the program meet your expectations?
5. Is there anything that the administration can do to support you in implementing some of what you learned?
6. Was there anything that you learned in the program that change other aspects in your life outside of your work? How readily are you to practice/put in place what you learned in your life?
7. What did you think about the mindfulness practices that was shared? For example, starting each session with mindfulness meditation.
8. What did you think about the weekly reflective journaling?

After the initial focus group, the leader reviewed the transcripts and provided initial summaries. The research team reviewed the transcripts and noted reoccurring themes. The leader did a second round of review of transcripts to identify and code related quotes or discrepancies after initial team review. Once the second review and coding were completed, the research team came together to review discrepancies and reach consensus of group summary. After the second round of team discussion, themes were revised and finalized.

### 2.8. Statistical Analysis

Summary statistics (ranges, means, standard deviations, frequencies, and missing rates) were computed for data collected at pre-test and at post-test. Demographic data missing at pre-test (for Cohort 1 only) were imputed from data provided at post-test when appropriate; this occurred for one participant missing age at pre-test, one participant missing income level at pre-test, and one participant missing years of employment at pre-test (no participant missed more than one). The only missing data on outcome measures were for minutes of physical activity per day in the past week reported at pre-test (n = 5 from Cohort 1), which constrained our analytic sample for that analysis.

Paired *t*-tests were used to determine whether there was, on average, significant (*p* < 0.05) change in scores from pre- to post-test on the CAMS-R, FFMQ subscales, minutes of physical activity per day from the Lifestyle Questionnaire, ProQOL subscales, and Perceived Cohesion Scale subscales (for Cohort 1 only); chi-square test was used to compare frequencies across categorical responses for servings of fruits and vegetables as well as sleep quality. Sensitivity analysis for the paired *t*-tests indicates that, with a sample of 19 and 15 matched pairs and α = 0.05, we had 80% power to detect a medium effect (dz = 0.59 and 0.68), which corresponds to a change of approximately half a standard deviation (SD) from the pre-test mean.

## 3. Results

### 3.1. Sample Demographics (Table 1)

In Cohort 1, the 19 female nurse managers were on average in their mid-40s, and most were married. Approximately half were White and the other half Black (one marked race as Other and did not specify, one marked race as Other and wrote in Caribbean). The vast majority reported speaking English at home, and all owned the home they were living in with 1–5 household members. The group was evenly split between having a bachelor’s degree or a graduate degree and between having an annual household income of USD 100,00–150,000 or over USD 200,000. The majority of households relied primarily on the nurse manager’s income. The majority of participants worked in the nursing field for at least 16 years.

**Table 1:** Sample demographic characteristics.

	Cohort 1 (n = 19)		Cohort 2 (n = 17)	
	M (SD)	% (n)	M (SD)	% (n)
Age in years	43.8 (9.0)		35.7 (10.9)	
Relationship status				
Never married/single		21.1 (4)		52.9 (9)
Living with partner		10.5 (2)		17.6 (3)
Married		68.4 (13)		11.8 (2)
Divorced/widowed		0		17.7 (3)
Race				
White, % (n)		47.4 (9)		0
Black, % (n)		42.1 (8)		100 (17)
Other, % (n)		10.5 (2)		0
Household language English		94.7 (18)		100 (17)
Number in household	2.95 (1.31)		3.3 (1.3)	
Education				
High school or Associate’s degree		0		47.0 (8)
Bachelor’s degree		47.4 (9)		35.3 (6)
Some graduate school		5.2 (1)		17.6 (3)
Graduate degree		47.4 (9)		0
Income level				
Less than USD 50,000		0		76.5 (13)
USD 50,000–75,000		0		17.6 (3)
>USD 75,000		0		5.9 (1)
USD 100,000–125,000		10.5 (2)		
USD 125,000–150,000		36.8 (7)		
USD 150,000–175,000		5.3 (1)		
USD 175,000–200,000		5.3 (1)		
More than USD 200,000		42.1 (8)		
Primary income source				
Salary/wage from own job		68.4 (13)		82.4 (14)
Salary/wage from another household member’s job		26.3 (5)		17.6 (3)
Years employed in current job	15.8 (6.2)		2.4 (5.9)	
Years working in the field				
Less than 1				11.8 (2)
1–3 years				23.5 (4)
4–7 years		10.5 (2)		29.4 (5)
8–10 years		0		5.9 (1)
11–15 years		15.8 (3)		17.6 (3)
16–20 years		36.8 (7)		11.8 (2)
More than 20 years		36.8 (7)		0

Among the 17 ECEs in Cohort 2, all were African American females who primarily speak English at home. The majority were single/never married and renting their apartment or in public housing. Half had less than a college education and incomes less than USD 50,000 annually. Two-thirds had been working in the early education field for less than 10 years.

### 3.2. Outcome Analyses (See Table 2)

According to paired *t*-tests, this sample of nurse managers significantly improved on the ‘Describing’ and ‘Non-judging of inner experience’ aspects of mindfulness from before to after this intervention. On the Lifestyle Questionnaire, the only significant change was on improved self-reported sleep quality, which, at post-test, almost all reported as fairly or very good. On the ProQOL, there was a significant decline in ‘Compassion Fatigue’. There was also significant improvement in both ‘Belonging’ and ‘Morale’ subscales of the Perceived Cohesion Scale.

**Table 2:** Change in measures from pre- to post-test for Cohort 1 (n = 19).

	Pre-Test M (SD)	Post-Test M (SD)	Average Change	Paired *T*-Test
Five Facet Mindfulness Questionnaire				
Observe	28.32 (4.58)	29.53 (3.99)	1.21 (5.19)	1.02
Describe	27.68 (5.01)	29.53 (5.03)	1.84 (3.34)	2.41 *
Acting with awareness	27.0 (5.71)	28.8 (4.95)	1.79 (3.84)	2.03
Non-judging of inner experience	26.47 (5.69)	29.37 (4.56)	2.89 (3.53)	3.58 **
Non-reactivity to inner experience	21.89 (3.49)	23.63 (3.68)	1.74 (3.8)	1.99
Professional Quality of Life Scale				
Compassion satisfaction	36.68 (7.07)	38.74 (6.88)	2.05 (6.89)	1.30
Burnout	23.84 (6.18)	22.11 (4.61)	−1.74 (4.29)	−1.76
Compassion fatigue	24.21 (5.69)	21.16 (5.79)	−3.05 (5.09)	−2.61 *
Perceived Cohesion Scale				
Belonging	15.68 (2.80)	17.84 (2.91)	2.16 (2.69)	3.49 **
Morale	15.84 (2.46)	17.58 (2.87)	1.74 (2.35)	3.22 **
Lifestyle Questionnaire				
Minutes/day in physical activity over past week	46.43 (38.55)	54.64 (56.47)	8.21 (40.16)	0.77
	% (n)	% (n)		χ^2^
Servings of fruit in typical day				17.92
0	20 (4)	5 (1)		
1	25 (5)	30 (6)		
2	25 (5)	35 (7)		
3	10 (2)	15 (3)		
4 or more	15 (3)	10 (2)		
Servings of vegetables in typical day				12.57
0				
1	21 (4)	5 (1)		
2	42 (8)	42 (8)		
3	15 (3)	42 (8)		
4 or more	21 (4)	10 (2)		
Sleep quality in past week				14.99 *
Very good	10 (2)	15 (3)		
Fairly good	42 (8)	74 (14)		
Fairly bad	42 (8)	10 (2)		
Very bad	5 (1)			

*Note. p*-values is indicated as * *p* < 0.05, ** *p* < 0.01.

For Cohort 2, according to paired *t*-tests, this sample of ECEs significantly decreased in terms of cognitive and affective mindfulness (see Table 3). No other pre-post comparisons for healthy lifestyle or professional quality of life were significant.

### 3.3. Qualitative Results

The qualitative data gathered from program participants and staff involved in the implementation of CPR^2^ revealed three overarching themes: group structure, purpose, and overall experience. These themes represent the immediate experiences and reflections of participants regarding the program’s delivery and its initial impact. They were identified through discussions with group participants and feedback from program directors and other staff who supported the program’s implementation.

### 3.4. Group Structure

When asked about satisfaction with group structure (i.e., length of time, schedule of groups, etc.), most participants felt the one-hour time was sufficient, while others believed an additional 15 min grace in the beginning would be helpful to allow for login difficulties and check-ins. Participants shared that the group would be better in person and recommended additional sessions. Despite being in the virtual space, one participant described: “*this was a really productive group of women, and I was really proud of all of us, I couldn’t imagine what this would be like in person*”. Additionally, participants emphasized that the breakout groups helped to “*create more meaningful connections*”.

### 3.5. Purpose

Participants shared that the program components, specifically mindfulness practices, journal prompts and mood rating check-in/out were “*eye-opening*” and “*good energy releases*”. Additionally, participants incorporated learnings from the group into their personal and professional routines, with one participant reflecting, “*It gave me permission to think about myself a little more…forgive myself a little bit more*”. Mindfulness practices were particularly helpful as one participant stated “*…[mindfulness practices] help center [us] for the hour*”. Another found, “*the rainbow meditation with the colors was short and easy…I do it at home*”, *and* “*I like the [journal] prompts; it was useful to get me thinking*”. Furthermore, when discussing the mindful movement session, one participant shared that “*…dancing was a different type of energy release…you were able to let yourself go and it was a good form of release…I do it with my daughters*”. Furthermore, with regard to the program’s topics the participants reported significant learnings and takeaways. Participants walked away with several skills to incorporate into their lives and were encouraged to share this with their families, friends, and colleagues. One group participant stated, “*I didn’t say a lot during the group, but I did soak a lot of it in. I used a lot of it. It really helps me have a sense of peace*”.

### 3.6. Overall Experience

The benefits of the small group format were highlighted by the participants. A participant said, “you can come in these sessions feeling the work, but just being here just makes you feel relaxed…you guys really become my sisters…it’s a judgement free zone and I really appreciate you”. One group member stated, “I’m not really the sort of person that likes to [vent] in a large group…it was difficult; but it was also nice to have that sense of security, of that safe space”. A noted mood shift was observed by several, “[We] came in a very hectic mind frame and then after 10 or 15 min that really started to change”. After reflecting on the impact of the group, a member said, “We only got to the tip of the iceberg, and even at the tip of the iceberg we really achieved something”. Another participant stated, “so many techniques we can use in different areas, like the Johari’s window…I like the tangible stuff that I can reuse and takeaway”. This echoes other participants who stated that they would benefit from an expanded program. Through the CPR^2^ participation, group members were further able to engage in self-reflection and stated that the program, “…gave [them] permission to think about [themselves] a little more…forgive [themselves] a little bit more”. Lastly, the participants agreed that they “…see the value [of CPR^2^ program] for our staff” and were “really grateful for the opportunity”. At the conclusion of the program, one participant noted, “…I [wished] it was something [they] could do regularly, and it was embedded in [their] culture”, further emphasizing the need for small groups for providers focused on improving their wellbeing. Participants expressed profound appreciation for the program’s focus on their wellbeing, often highlighting the emotional and professional challenges they face in their caregiving roles. One participant eloquently captured this sentiment, stating: “This group has been fulfilling in a way I did not even expect to need. I work in an industry…all of these ladies’ work in an industry where we’re often overlooked, forgotten, overworked, and underthought about. It’s refreshing to see [a program] pour into us what we pour into students, clients, every single day of the week. And we don’t expect a thank you, but your pursuit of us and your pour into us was thank you enough”.

## 4. Discussion

This study sought to explore the feasibility and acceptability of a group-based wellness intervention tailored for nurse managers and early childhood educators (ECEs). The program hypothesized that participants would integrate mindfulness practices into their daily lives and adopt healthier behaviors, leading to measurable reductions in compassion fatigue and burnout. By targeting two distinct cohorts—nurse managers in healthcare settings and ECEs in early childhood education environments—this research aimed to assess how a wellness program could address the unique challenges faced in each context. Nurse managers in healthcare settings often experience high levels of stress and burnout due to demanding work conditions, including long hours, emotional strain, and administrative pressures [80,81,82]. Similarly, ECEs face unique stressors, such as fostering the developmental growth of children, addressing the emotional needs of both students and their families, and maintaining a positive classroom environment while working for relatively low pay in high-turnover environments [33,50]. By utilizing a two-cohort design, CPR^2^ explored the diverse workplace contexts that shape the emotional capacities of nurse managers and ECEs, emphasizing the importance of both individual emotional capacity and workplace dynamics in fostering professional wellbeing. This design allowed for a deeper understanding of the specific stressors and supports unique to each group.

The findings from this study underscore the nuanced outcomes of the CPR^2^ intervention across two distinct cohorts, reflecting both its impact and the differing baseline characteristics of the groups. For the nurse managers in Cohort 1, significant improvements were observed in the mindfulness dimensions of “*Describing*” and “*Non-judging of inner experience*”. These changes suggest an enhanced ability to articulate and accept emotional states without criticism, key skills for navigating the high-stress environments of healthcare. Furthermore, a significant reduction in *Compassion Fatigue*, as measured by the ProQOL, highlights the intervention’s potential to alleviate one of the most pressing challenges in nursing, the emotional burden associated with caregiving. While there was a slight decrease in burnout, this change was not statistically significant, suggesting a trend towards improvement but not a definitive outcome. In contrast, there were significant improvements in both subscales of the Perceived Cohesion Scale, suggesting that the program fostered stronger connections and a sense of belonging among participants. These findings align with the idea that group-based interventions can improve social connectedness and collective morale, which are critical for reducing burnout and fostering a positive work environment [48,83]. Additionally, the results are particularly significant in the context of the high-stress environments in which nurse managers operate, where feelings of isolation and a lack of social support can exacerbate burnout and stress [49].

In contrast to the ProQOL results for the nurse managers, ECEs showed no significant improvements across the same subscales. Their compassion satisfaction, burnout, and compassion fatigue scores remained relatively stable, with none of the changes reaching statistical significance. These findings suggest that while the CPR^2^ program had some positive effects for nurse managers, particularly in reducing compassion fatigue, it did not achieve comparable results for ECEs. This discrepancy may reflect the unique challenges faced by ECEs, such as low wages, high turnover, and the demands of managing children’s developmental needs, which may require more tailored or intensive interventions to address [26,50]. Additionally, the contrasting results between the two cohorts emphasize the importance of contextual factors, such as organizational support and workload, in influencing the effectiveness of wellness programs [44,45,46,47,57,58,66].

Regarding mindfulness, participants in the early childhood settings showed a significant decrease in cognitive and affective mindfulness. It is possible that the CPR^2^ program, which incorporates mindfulness-based activities, may have encouraged participants to become more aware of their emotions and thoughts initially, but over time, as they practiced mindfulness, their ability to regulate these emotions and thoughts might have increased, leading to a lower score on this scale. This might also reflect adaptation or a shift in how participants process their emotional experiences, indicating an improvement in emotional regulation or coping strategies [48,83]. This finding could also suggest that the intervention’s specific mindfulness components may have influenced participants’ cognitive and affective awareness in ways that are not directly aligned with traditional measures of mindfulness.

Mindfulness interventions often intersect with cultural dynamics, which can shape their application and impact within specific populations [83]. In their study, Bermudez et al. [83] examined the impact of mindfulness-based interventions on chronic pain and identified significant differences in treatment responses across racial groups. Specifically, they found that Black participants reported greater improvements in emotional regulation and perceived stress compared to their White counterparts. However, Black participants were less likely to report reductions in pain intensity, highlighting potential differences in how mindfulness interventions address physical versus emotional dimensions of chronic pain across racial groups [84]. Research highlights that mindfulness can mitigate the physiological and psychological effects of racial trauma by promoting emotional regulation, reducing hypervigilance, and creating a space for reflection and self-compassion [85,86]. Additionally, culturally tailored mindfulness approaches that incorporate elements of community connection, spirituality, and cultural affirmation have been shown to deepen engagement and effectiveness for Black participants, addressing both individual and collective stressors [87]. This adaptation in how mindfulness is experienced and applied could lead to shifts in how emotional regulation and cognitive awareness are measured, potentially reflecting a deeper, culturally specific adaptation rather than a simple decrease in mindfulness [88,89,90,91,92,93].

In contrast, both nurse managers and ECEs showed significant improvement in sleep quality, as measured by the Lifestyle Questionnaire, with a notable improvement at post-test. This change is particularly relevant given the critical role of sleep in overall wellbeing and the ability to manage stress and burnout. This aligns with evidence that mindfulness practices can positively influence sleep patterns by reducing stress and promoting relaxation. Research has consistently demonstrated that sleep quality is closely linked to emotional regulation and the ability to perform in high-stress environments [94,95,96,97]. This finding suggests that CPR^2^ may help address one of the key factors contributing to burnout and compassion fatigue among frontline workers by improving their sleep quality, which in turn supports their capacity to manage the emotional demands of their roles. Improved sleep quality could also be indicative of better emotional regulation and a reduction in stress, both of which are essential for enhancing ‘resilience’ in demanding work environments [49,96,97]. The fact that both groups, despite their distinct work contexts, reported improvements in sleep quality further underscores the potential of CPR^2^ to positively impact critical aspects of wellbeing across diverse populations, from healthcare professionals to early childhood educators. This result aligns with previous studies that have highlighted the importance of addressing sleep in wellness interventions for frontline workers [96,97]. These improvements in sleep quality contribute to the growing body of evidence supporting the feasibility and impact of group-based wellness interventions in high-stress environments.

Building on the three themes identified in the results, the discussion explores five actionable insights, referred to as the ‘*Keys to Success*’, which are critical factors for enhancing the program’s impact and sustainability. These strategies—identifying an onsite ‘Wellness Champion’, assessing organizational readiness, facilitating leadership wellness sessions, maintaining flexibility to address organizational challenges, and fostering peer support—are derived from both qualitative data and interpretation of participants’ and staff members’ reflections on program implementation.

**Identifying an Onsite “Wellness Champion”:** Identifying an onsite *Wellness Champion* is a key strategy for embedding systemic support for staff wellbeing and ensuring that wellness programs are aligned with both organizational culture and staff needs. Recognizing the impact of appointing a dedicated individual who is not only committed to staff personal wellbeing but also empowered to champion mental health and wellbeing at the organizational level is vital. The *Wellness Champion* serves as a bridge between the CPR^2^ facilitator and the organization, ensuring that the goals of the program are tailored to the specific needs and culture of the workplace. By having a “champion” embedded within the organization, the program can more effectively address real-time challenges and adapt its content to meet the evolving needs of the staff. This approach not only enhances the program’s relevance but also fosters a sense of ownership and accountability within the organization. Ultimately, this strategy increases the likelihood of sustained engagement with wellness practices and promotes long-term integration into the organizational framework [48,98,99].

**Assessing Onsite Readiness:** Assessing onsite readiness is a crucial factor in the successful implementation of the CPR^2^ program. This assessment involves a comprehensive evaluation of the organizational culture and climate, existing wellness initiatives, and the availability of “protected time” for staff to engage in wellness programming. Organizational culture, which encompasses the values, activities, and philosophy that define an organization’s identity, often shapes how employees interact with one another and those they serve [99,100,101]. Cultural climate, on the other hand, reflects the emotional tone or feeling within the workplace [100,101,102,103,104,105]. Both organizational culture and climate have been shown to significantly influence job satisfaction, stress levels, and overall wellbeing [48,49].

Understanding the baseline readiness of the site allows for the tailoring of the program’s structure and content to better address the specific needs and challenges of the organization. For example, this could include considering the unique characteristics of the populations participating in the program, such as ECEs versus nurse managers, and determining the most effective logistics for the group (e.g., program timing, provision of meals, CEUs or payment for completion). Identifying potential barriers to implementation, such as time constraints or limited resources, is essential for ensuring the program’s success. Proactively addressing these barriers can be the key to successful program completion. Conducting a readiness assessment ensures that the program is not only relevant but also feasible within the specific organizational context, ultimately increasing the likelihood of its success.

**Facilitating Leadership Wellness Sessions:** Facilitating leadership wellness sessions was identified as a key strategy for the success of the CPR^2^ program. Leadership buy-in is crucial, as leaders play a pivotal role in shaping organizational culture. Their commitment to wellness initiatives can directly influence staff engagement and participation [102,103]. By offering wellness sessions specifically for leadership, organizations can cultivate a culture of wellbeing from the top down. These sessions equip leaders with the tools and strategies necessary to manage their own stress and burnout, thus enhancing their capacity to support their teams effectively [49,81,82]. Furthermore, when leaders actively engage in wellness programs, it can foster increased buy-in from other leadership members and improve overall program implementation, reinforcing the organizational commitment to staff wellbeing [48,82,101].

**Maintaining Flexibility to Address Organizational Issues:** Maintaining flexibility is a cornerstone of effective program implementation, as it enables interventions like CPR^2^ to respond dynamically to the challenges presented by real-world settings. For instance, one of the CPR^2^ cohorts experienced a significant disruption when the global pandemic forced an abrupt shift from in-person sessions to fully virtual formats. This highlighted the critical need for flexibility in responding to unforeseen crises and ongoing organizational challenges, such as staffing shortages, changes in organizational priorities, and varying levels of participant engagement [101]. The program’s ability to adjust its schedule, content, and delivery methods allowed it to remain both relevant and effective during this disruption.

Flexibility ensures the program’s alignment with the evolving needs of both participants and organizations. By incorporating participant feedback and adapting to the changing organizational landscape, CPR^2^ can maintain its effectiveness even in the face of unexpected challenges. This adaptability helps the program remain relevant to its target population, supporting sustained engagement and long-term outcomes [95,101]. Ultimately, by ensuring the program remains attuned to both individual and organizational goals, CPR^2^ maximizes its potential impact, fostering a culture of wellbeing that is responsive to the unique needs of each environment.

**Sustainability and Co-Creation:** Sustainability in workforce wellness hinges on a co-creation framework, where the perspectives of participants are meaningfully integrated alongside those of organizational leaders and program facilitators. This inclusive and equitable approach ensures that initiatives like CPR^2^ are not merely top-down directives but are collaboratively shaped to address the real challenges and lived experiences of the workforce. By actively involving participants in the design and implementation phases, wellness programs can be tailored to resonate with the unique culture and needs of each workplace, significantly enhancing their relevance and effectiveness.

This collaborative process fosters greater participant engagement by giving individuals a tangible stake in a program that directly reflects their input and priorities. It also promotes transparency and trust, as participants see their contributions actively influencing the program’s development. Such an approach not only strengthens individual commitment but also aligns wellness strategies with organizational goals, creating a cohesive and adaptable framework.

Ultimately, co-creation serves as a catalyst for systemic change, bridging individual wellbeing with broader organizational health. By integrating participant voices into the core of wellness initiatives, programs like CPR^2^ can achieve both meaningful impact and sustainable results, fostering a culture of continuous growth and support at all levels.

In the case of CPR^2^, understanding the specific organizational culture and climate of the work environments in which nurse managers and ECEs operate was essential for the program’s success. This recognition allowed the intervention to be tailored to the unique schedule needs of each cohort, ensuring that strategies for stress management, emotional regulation, and peer support are aligned with the organizational context. Moreover, a program that integrates organizational support—such as leadership involvement, readiness assessments, and a Wellness Advocate—has a better chance of promoting sustainable change and fostering a healthier organizational climate [98,99,100,101,102]. To enhance the effectiveness of the program, we worked closely with leadership at each site to understand the specific needs and challenges. Both the nurse managers and ECEs were given protected time by leadership to participate in the program, which contributed significantly to its feasibility and success. By prioritizing protected time and leadership support, CPR^2^ was better able to foster a supportive and sustainable work environment that aligned with the goals of the intervention.

## 5. Limitations and Future Directions

This study has several limitations that should be considered when interpreting the findings and planning future iterations of the program. First, the relatively small sample size and the inclusion of only two workplace contexts—nurse managers and ECEs—may limit the generalizability of the results to other occupational groups. Additionally, the study’s participating centers, one focused on domestic violence survivors and the other on a homeless population, limit the generalizability of findings to typical early childhood education settings. The unique challenges faced by these populations could have influenced outcomes differently compared to more conventional ECE environments. Future research should include a more diverse range of workplaces and roles to better understand how CPR^2^ can be adapted across various settings. Second, barriers to participation, such as low attendance in focus groups and logistical challenges like scheduling conflicts, underscore the need for refined engagement strategies. Future iterations could explore hybrid delivery models or asynchronous program components to increase accessibility and flexibility. Lastly, the groups were virtual due to COVID-19 pandemic, future iterations of the group may benefit from in-person meetings in order to bolster social cohesion further.

Both Cohort 1 and Cohort 2 followed the same intervention design, with both groups receiving the same mindfulness program. However, social cohesion was assessed differently for each cohort due to practical considerations during the data collection phase. Additionally, while the two-cohort design allowed for comparisons between distinct workplace contexts, the absence of a control group limits the ability to attribute observed changes directly to the intervention. While conventional research methods like surveys and interviews were used, it is important to acknowledge that these methods may not fully capture the complexities of participants’ experiences. Another limitation is the absence of a measure of organizational culture and climate. Organizational culture and climate play a critical role in shaping the emotional capacities and wellbeing of professionals, particularly in high-stress fields such as healthcare and early childhood education. Research indicates that the organizational environment—characterized by leadership style, workplace norms, and available resources—greatly influences how employees experience and respond to stress [98,103,104]. A supportive organizational culture, where employee wellbeing is prioritized and valued, can mitigate the emotional toll of demanding jobs, whereas a negative or neglectful climate can exacerbate burnout and emotional distress [98,105,106]. Future research should include assessments of organizational culture and climate to better understand their role in shaping the effectiveness of wellness interventions.

Furthermore, a more inclusive and diverse understanding of wellbeing, especially one rooted in cultural and community contexts, is being increasingly recognized as essential for addressing the unique needs of diverse populations. Integrating non-Western ways of knowing, such as storytelling, oral histories, and participatory action research (PAR), offers opportunities to explore wellbeing in ways that more contextually relevant and culturally resonant. Ethnographic methods and creative, art-based approaches like music or dance could also be valuable in capturing the nuanced ways in which cultural contexts influence wellness. The inclusion of music and dance in CPR^2^ generated a positive response and could be expanded in future studies.

Sustainability remains a critical area for further development. While the introduction of Wellness Champions and leadership engagement showed promise, their long-term impact was not assessed. Future research should explore longitudinal outcomes to determine whether CPR^2^’s benefits are sustained over time and identify factors that enhance or hinder its lasting integration into workplace culture. Future directions should also consider the application of CPR^2^ in hybrid work environments, particularly as workplace dynamics evolve post-pandemic. Co-creation with participants and leadership will be essential to ensure the program’s adaptability and ongoing relevance across diverse organizational settings.

## 6. Conclusions

This study contributes to the growing body of knowledge on wellness programs in high-stress occupations, particularly among nurse managers and early childhood educators. The CPR^2^ program demonstrated the potential to address both individual and organizational needs by fostering wellness practices that promote emotional regulation, mindfulness, and social connection in the workplace. The findings suggest that such interventions can create meaningful shifts in participants’ wellbeing, highlighting the importance of holistic, strengths-based approaches to wellness in work environments that face systemic stressors. Although the study’s scope was limited by sample size and methodological constraints, its findings underscore the relevance of integrated wellness programs in professions that involve high levels of emotional labor and caregiving. Building on the findings of this study, we are expanding our efforts to explore wellness in early childhood through a randomized controlled trial (RCT). This ongoing research will contribute valuable insights into the effectiveness of wellness interventions within early childhood settings, supporting the ongoing effort to enhance educators’ wellbeing. Through continued research and refinement, it is hoped that wellness interventions can be better tailored to meet the unique needs of diverse workforces, promoting long-term wellbeing and sustainable caregiving practices across various professional domains.

## Figures and Tables

**Table 3:** Change in measures from pre- to post-test for Cohort 2 (n = 17).

	Pre-Test M (SD)	Post-Test M (SD)	Average Change	Paired *T*-Test
Cognitive and Affective Mindfulness Scale	34.1 (6.2)	32.2 (5.6)	−1.9	1.94 *
Professional Quality of Life Scale				
Compassion satisfaction	42.5 (4.7)	41.9 (5.7)	−0.53	0.43
Burnout	19.6 (4.2)	20.7 (3.8)	1.1	0.78
Compassion fatigue	21.3 (3.8)	21.1 (4.9)	0.2	0.11
Perceived Cohesion Scale		29.6 (11)		
Lifestyle Questionnaire				
Minutes/day in physical activity over past week	54.4 (35.2)	56.0 (44.9)	1.6	0.12
	% (n)	% (n)		χ^2^
Servings of fruit in typical day				17.92
0	20 (4)	5 (1)		
1	25 (5)	30 (6)		
2	25 (5)	35 (7)		
3	10 (2)	15 (3)		
4 or more	15 (3)	10 (2)		
Servings of vegetables in typical day				12.57
0				
1	(4)	(1)		
2	(8)	(8)		
3	(3)	(8)		
4 or more	(4)	(2)		
Sleep quality in past week				14.99 *
Very good	11.8% (2)	6.7% (1)		
Fairly good	64.7% (11)	73.3% (11)		
Fairly bad	11.8% (2)	20% (3)		
Very bad	11.8% (2)	0		

*Note*. *p*-values is indicated as * *p* < 0.05.

## Data Availability

Data are contained within the article.

## Appendix A. Sessions Overview

CPR^2^ Mindfulness Session Topics, Learning Objectives, and Reflections

**(Session 1) Mindfulness and CPR^2^ Introduction. *Objectives (OBJ)*** (Sessions follow a repeating structure but with weekly topics, the following objects are included every week: Emotional self-reflection assessment “One Word Check-In”, Facilitators lead Mindfulness Practice, Revisit reflective journal assignments as a group, Practice “Reflective Journaling” as a group, and “Emotional self-reflection assessment “One Word Check-Out.”): Introduce CPR^2^ provider well-being work; develop group agreements; explore understanding of mindfulness practice; to learn differences between burnout, compassion fatigue, secondary traumatic stress, and vicarious trauma; to begin to explore self-care plan. ***Wellness Wheel Activity (WWA)****:* Use this activity to introduce domains of wellness; provider will identify personal domains of wellness and begin to explore self-care plan. ***Reflective Journaling Prompt (RJP):*** “How do you notice when you’re nearing burnout?”
**(Session 2) Mindfulness and Goal Setting. *OBJ***: Discuss goals from WWA; Introduce SMART Goals. ***Goal Setting Activity****:* wellness wheel activity put into practice; providers will set their individual SMART goals. ***RJP****:* “How can you use your accountability partners to support your goals?”
**(Session 3) Mindfulness and Cognitive Well-being. *OBJ****:* Discuss the connection between thoughts, feelings, and behaviors to cognitive well-being; Four Remembrance Practices. ***Cognitive Well-being Activity****:* Evaluate scenarios where their thoughts, feelings and behaviors may reflect self-judgement. The goal is for providers to answer “when I think [blank], I feel [blank], and I do [blank] to pause and evaluate their self judgement. ***RJP****:* “In moments of stillness, how do I see myself?”
**(Session 4) Mindfulness and Emotional Well-being: Stress. *OBJ****:* Discuss emotional bandwidth; Distinguish between thrive and survive emotions; Identify benefits to 8 survive emotions. ***Grounding Activity****:* Introduce 5 senses grounding technique, an application that providers can use to get emotionally centered. Incorporate breath techniques as a tangible, hands-on strategy. ***RJP***: “Reflect on your past week, how did thrive and survive emotions show up? Did you experience one more than the other?
**(Session 5) Mindfulness and Emotional Well-being—COVID and beyond. *OBJ****:* Discuss how stress and trauma show up in daily life; discuss coping. ***Coping Activity****:* Identify healthy alternatives to stress incorporate different grounding techniques explored earlier. ***RJP****:* What healthy coping response will you try when faced with this question: “When I feel stressed, I can…”
**(Session 6) Mindfulness and Physical Well-being—Sleep/Nutrition. *OBJ****:* Identify 5 techniques to 1) improve sleep and 2) mindful eating. ***Sleep Activity****:* Discuss how to foster pro-sleep habits. ***Nutrition Activity****:* Emotions and eating, full cycle review of “why, when, what, how, how much and where?” of eating habits. ***RJP***: Tonight, notice how it feels when you climb into bed. Notice what comes into your mind once you get situated in bed…Tomorrow morning, write down thoughts …
**(Session 7) Mindfulness and Physical Well-being—Activity and Movement. *OBJ****:* Discuss mindful movement and connection to wellness.***Movement activity**:* Providers participate in different movement activities. ***RJP****:* Mindful Walking by Tara Brach. Follow the activity, reflect/share experiences.
**(Session 8) Mindfulness and Social Well-being*. OBJ****:* Discuss interpersonal relationships and wellness; Introduce Johari Window; Introduce Relational Dialectics Theory (RDT). ***Johari Window Activity****:* Johari Window is used as a method for self-discovery; watch video/respond to questions; watch video on RDT. ***RJP****:* Participants will be provided with RDT video and four relational questions and asked to choose one for reflective journaling.
**(Session 9) Mindfulness and Self-Compassion*. OBJ****:* Discuss ways to build resilience; Explore self compassion; Identify self-care strategies; Discuss elements of trust (Braving; Brown). ***Self-Compassion Activity****:* Self-compassion activities are linked to self-care. Discuss the seven elements of trust (Braving). ***RJP****:* Go through Guided Meditation and reflect on the experience. RAIN Self-Compassion Guided Meditation by Tara Barch
**(Session 10) Mindfulness and Compassion to others—pulling it all together. *OBJ****:* Explore Compassion for others; Discuss the integration between all previous sessions. Close out program. ***Closing Program Activity****:* Encourage discussion on holistic program outcomes. Reflect as a group how individual week lessons relate to one another. ***RJP****:* Moving forward I will be committed to…

## Appendix B. Focus Group Protocol

Introduction:

Thank you for coming in to talk with us today. I want to start out by saying that everything you say in here will be kept confidential. We will only give summaries of the group’s opinions in order to improve the program for next time. Before we get started, are there any questions?

*[Poll in the beginning to see who can stay later. Norm that we have a lot to get through in 45 min and we need to be concise with our answers.]*

Today we are going to ask for your feedback on your experiences during the CPR^2^ Wellness Group you’ve just participated in.

1. What was it like for you to participate in this program?
  - PROBE: What was the most significant part the experience?
2. What did you think of the length of the *Educator Wellness* sessions?
  - PROBE: Would you have liked it to be longer? Shorter?
  - PROBE: Explore day/time of the program
3. What did you think of the number of sessions (10 sessions total)?
  - Would you have liked more sessions or fewer?
  - PROBE: Did the group stop at a good time? Did it feel complete?
4. If we were to do another round of *Educator Wellness*, is there anything you would like to see done differently?
  - PROBE: Anything you would change? Add? Takeaway?
5. Is there anything that the administration can do to support you in implementing some of what you learned in *Educator Wellness*?
  - PROBE: Specific examples?
6. Did the things you learned in the program change other things in your life outside of your work?
  - PROBE: Your sleep? Relationships with others in your life? Health?
  - PROBE: How did it affect your stress levels
  - PROBE: Did it provide any insights about yourself?
7. What did you think about the mindfulness practices that was shared? For example, starting each session with mindfulness meditation.
  - PROBE: Would you have liked more? Fewer?
  - PROBE: What did you think about the Mindfulness Quote of the Week?

Closing Question

1. Is there anything else you would like to share that we haven’t already discussed?
